# Genetic mutations in HER2-positive breast cancer: possible association with response to trastuzumab therapy

**DOI:** 10.1186/s40246-023-00493-5

**Published:** 2023-05-18

**Authors:** Nermine H. Zakaria, Doaa Hashad, Marwa H. Saied, Neamat Hegazy, Alyaa Elkayal, Eman Tayae

**Affiliations:** 1 Department of Clinical and Chemical Pathology, Faculty of Medicine, University of Alexandria, Alexandria, Egypt; 2 Department of Clinical Oncology and Nuclear Medicine, Faculty of Medicine, University of Alexandria, Alexandria, Egypt

**Keywords:** HER2-positive breast cancer, Genetic variants, NGS, Trastuzumab, Drug resistance

## Abstract

**Background:**

HER2-positive breast cancer occurs in 15–20% of breast cancer patients and is characterized by poor prognosis. Trastuzumab is considered the key drug for treatment of HER2-positive breast cancer patients. It improves patient survival; however, resistance to trastuzumab remains a challenge in HER2-positive breast cancer patients. Therefore, the prediction of response to trastuzumab is crucial to choose optimal treatment regimens. The aim of the study was to identify genetic variants that could predict response to anti-HER2-targeted therapy (trastuzumab) using next-generation sequencing.

**Method:**

Genetic variants in the hotspot regions of 17 genes were studied in 24 Formalin-Fixed Paraffin-Embedded (FFPE) samples using Ion S5 next-generation sequencing system. FFPE samples were collected from HER2‑positive breast cancer patients previously treated with anti‑HER2‑targeted treatment (Trastuzumab). Patients were divided into two groups; trastuzumab-sensitive group and trastuzumab-resistant group based on their response to targeted therapy.

**Results:**

We identified 29 genetic variants in nine genes that only occurred in trastuzumab-resistant patients and could be associated with resistance to targeted therapy including *TP53, ATM, RB1, MLH1, SMARCB1, SMO*, *GNAS*, *CDH1*, and *VHL.* Four variants out of these 29 variants were repeated in more than one patient; two variants in *TP53*, one variant in *ATM* gene, and the last variant in *RB1* gene. In addition, three genes were found to be mutated only in resistant patients; *MLH1, SMARCB1 and SMO* genes. Moreover, one novel allele (c.407A > G, p. Gln136Arg) was detected within exon 4 of *TP53* gene in one resistant patient.

**Conclusion:**

NGS sequencing is a useful tool to detect genetic variants that could predict response to trastuzumab therapy.

**Supplementary Information:**

The online version contains supplementary material available at 10.1186/s40246-023-00493-5.

## Introduction

Breast cancer is the most common cancer and the first cause of cancer deaths in females worldwide [1]. In 2020, there were 2.26 million newly diagnosed cases accounting for 24.5% of total new cancer cases, and 684,996 deaths of breast cancer accounting for 15.5% of total cancer deaths globally [1]. In Egypt, breast cancer was responsible for 32.4% of all newly diagnosed cancer cases and 10.3% of all cancer-related fatalities in 2020 [1].

HER2 overexpression occurs in approximately 15–20% of breast cancer cases [2]. HER2-positive breast cancer is characterized by a high histological grade, a high risk for metastasis, and therefore a worse prognosis [3]. However, anti‑HER2‑targeted drugs, such as trastuzumab, pertuzumab, and lapatinib, can block HER2 activity reducing tumor aggressiveness and improving patient survival [4]. They are typically given with chemotherapy as neoadjuvant or adjuvant treatment for HER2-positive breast cancer [5].

The response to anti-HER2-targeted therapy was found to vary among patients with the presence of patients who relapse or develop metastasis during therapy [6]. Therefore, prediction of response to HER2-targeted therapy is crucial to avoid undesirable side effects and for choosing more effective alternatives for patients [7].

HER2-positive breast cancer results from the interplay between genetic and lifestyle/environmental risk factors [8]. Genetic determinants can explain the resistance of some patients to anti-HER2 therapy [9]. Many genetic mutations in HER2 downstream signaling pathways were identified to confer drug resistance as mutations in *PI3K, Akt,* and *PDK* genes [10]. Mutations in DNA damage repair pathways were also investigated for association with treatment response such as *PTEN, TP53, ATM, STK11,* and *RB1* [11–15].

Therefore, studying genetic variants in tissue samples of breast cancer patients will aid in individualizing therapy with better outcomes [9]. Next-generation sequencing allows multiple parallel sequencing of several genes at same time [16]. While genome-wide analysis has the most significant role in the classification of breast cancer, targeted sequencing gives deeper coverage through reducing the number of analyzed genes [17, 18]. Targeted sequencing can be used to investigate hotspot cancer-driver mutations in breast cancer and thereafter study mutations that affect signaling pathways conferring anti-HER2 drug resistance [11, 19]. Therefore, we aimed in this study to survey genetic variants in HER2-positive breast cancer patients that may be associated with anti-HER2 drug (trastuzumab) resistance.

## Subjects and methods

This study is a retrospective study, in which formalin-fixed paraffin-embedded tissues (FFPE) samples were collected between December 2020 and December 2021 from 24 HER2‑positive breast cancer patients treated with anti‑HER2‑targeted therapy (trastuzumab), after approval of Alexandria Ethics Committee of Faculty of Medicine. The patients were recruited from Clinical Oncology and Nuclear Medicine Department at Alexandria Main University Hospital. Cases were divided into two groups; trastuzumab-sensitive group and trastuzumab-resistant group. Trastuzumab-sensitive group included 12 patients in complete remission for 2 years or more from the start of anti-HER2-targeted therapy. Trastuzumab-resistant group included patients who relapsed or developed metastasis during receiving or within 2 years of the targeted therapy. Patients with metastatic breast cancer at the time of diagnosis were excluded from the study. The positivity of HER-2 neu status was determined using immunohistochemistry (IHC) and fluorescence in situ hybridization. IHC was performed on paraffin-embedded tissue samples to evaluate hormone receptor (HR); estrogen (ER) and progesterone (PR). Informed consents were obtained from all enrolled patients in the study.

### DNA extraction

DNA was extracted from FFPE tissue samples using QIAamp DNA FFPE Tissue Kit (QIAGEN, Germany). The concentration of DNA was determined using Qubit™ 1X dsDNA HS (High Sensitivity) Assay with Qubit™ 4 Fluorometer (ThermoFisher Scientific, USA) according to the manufacturer’s recommendations.

### Library preparation

DNA libraries were constructed from 10 ng genomic DNA per sample using the Ion AmpliSeq™ Library Kit Plus (ThermoFisher Scientific, USA) according to the manufacturer’s protocol to study approximately 1500 COSMIC mutations from 17 oncogenes and tumor suppressor genes (*ATM, RB1, MLH1, NPM1, STK11, CDKN2A, TP53, SMARCB1, VHL, CDH1, EZH2, IDH1, IDH2, GNA11, GNAS, GNAQ,* and* SMO*)*.*

Ion Xpress™ Barcode Adapters Kit (Thermo Fisher Scientific, USA) and Agencourt™ AMPure™ XP Reagent (Beckman Coulter, USA) were used for amplicons adaptors ligation and purification to ensure that each individual sample had a unique ID. The final amplicon libraries were quantified using Ion Library TaqMan™ Quantitation kit (Thermo Fisher Scientific, USA) according to the manufacturer's instructions and were equalized to ~ 100 pM and then combined to form one library pool.

### Emulsion PCR and sequencing

The Ion 520™ and Ion 530™ Kit—OT2 (Thermo Fisher Scientific, USA) was first used to prepare enriched, template-positive Ion Sphere™ Particles (ISPs) using the Ion OneTouch™ 2 System (Thermo Fisher Scientific, USA) according to the manufacturer’s protocol. The enriched, template-positive ISPs were then loaded on Ion 520™ Chip (ThermoFisher Scientific, USA) and sequenced using Ion S5™ next generation sequencing system (ThermoFisher Scientific, USA).

### Bioinformatic analysis

Torrent Suite™ Software (ThermoFisher Scientific, USA) was used to plan and monitor sequencing runs, view sequencer activity, and analysis of barcode reads, alignment of reads to hg19 reference genome; and generation of run metrics to determine the quality of the run. Ion Reporter™ Software (ThermoFisher Scientific, USA) was used for the annotation of single-nucleotide, insertions, deletions, and splice site alterations. All genetic variants with a minimum depth of coverage of 30 × were included in the study. Allelic frequency ranged from 1.01 to 11.6%.

### Statistical analysis of the data

Data were analyzed using IBM SPSS software package version 20.0*.* (Armonk, NY: IBM Corp). Qualitative data were described using number and percent. Chi-square test was used for categorical variables, to compare between different groups. Fisher’s Exact or Monte Carlo correction for chi-square was used when more than 20% of the cells have an expected count less than 5. The Shapiro–Wilk test was used to verify the normality of distribution. Quantitative data were described using range (minimum and maximum), mean ± standard deviation. Student t test was used for normally distributed quantitative variables, to compare between two studied groups. Significance of the obtained results was judged at the 5% level.

## Results

### Patient characteristics

The age of trastuzumab-sensitive HER2-positive breast cancer patients ranged from 35 to 61 years old, while age ranged from 36 to 60 years old in trastuzumab-resistant group. The predominant histopathological subtype of breast cancer was infiltrating ductal carcinoma (100% of patients). Meanwhile, ER−, PR−, HER2+ breast cancer was the most common molecular subtype in both sensitive group and resistant group; representing 50% and 66.7% of patients, respectively. No significant difference was found between both groups as regards age, tumor stage, grade and lines of treatment. All demographic and clinicopathological data are summarized in Table 1.

**Table 1:** Clinicopathological data for HER2‑positive breast cancer patients

Parameters	Trastuzumab-sensitive cases (*n* = 12)	Trastuzumab-resistant cases (*n* = 12)	*p*
Age (years) at initial diagnosis,			
Min–max	35.0–61.0	36.0–60.0	0.837
Mean ± SD	49.42 ± 9.35	50.17 ± 8.31	
Hormone receptor status at diagnosis			
ER−, PR+, HER2+	1 (8.3%)	0 (0%)	0.521
ER−, PR−, HER2+	6 (50%)	8 (66.7%)	
ER+, PR−, HER2+	0 (0%)	1 (8.3%)	
ER+, PR+, HER2+	5 (41.7%)	3 (25%)	
TNM stage			
I	2 (16.7%)	0 (0%)	0.365
II	6 (50%)	5 (41.6%)	
III	4 (33.3%)	7 (58.4%)	
Grade			
Grade I	0 (0%)	0 (0%)	1
Grade II	6 (50%)	6 (50%)	
Grade III	6 (50%)	6 (50%)	
Clinical course			
Disease free	12 (100%)	0 (0%)	
Local relapse	–	5 (41.7%)	
Non-visceral metastasis (bone)	–	4 (33.3%)	
Visceral metastasis (lung and brain)	–	2 (16.7%)	
Death	–	1 (8.3%)	
Lines of therapy			
Anti-HER2-targeted therapy			
Trastuzumab (herceptin)	12 (100%)	12 (100%)	1.000
Chemotherapy: Doxorubicin (adriamycin)/cyclophosphamide, Paclitaxel (taxol), and/or Docetaxel (taxotere)	12 (100%)	12 (100%)	1.000
Hormonal therapy	6 (50%)	4 (33.3%)	0.680

*p* p value for comparing between the two studied groups

### Genetic variants in HER2-positive breast cancer patients

**Fig. 1:**
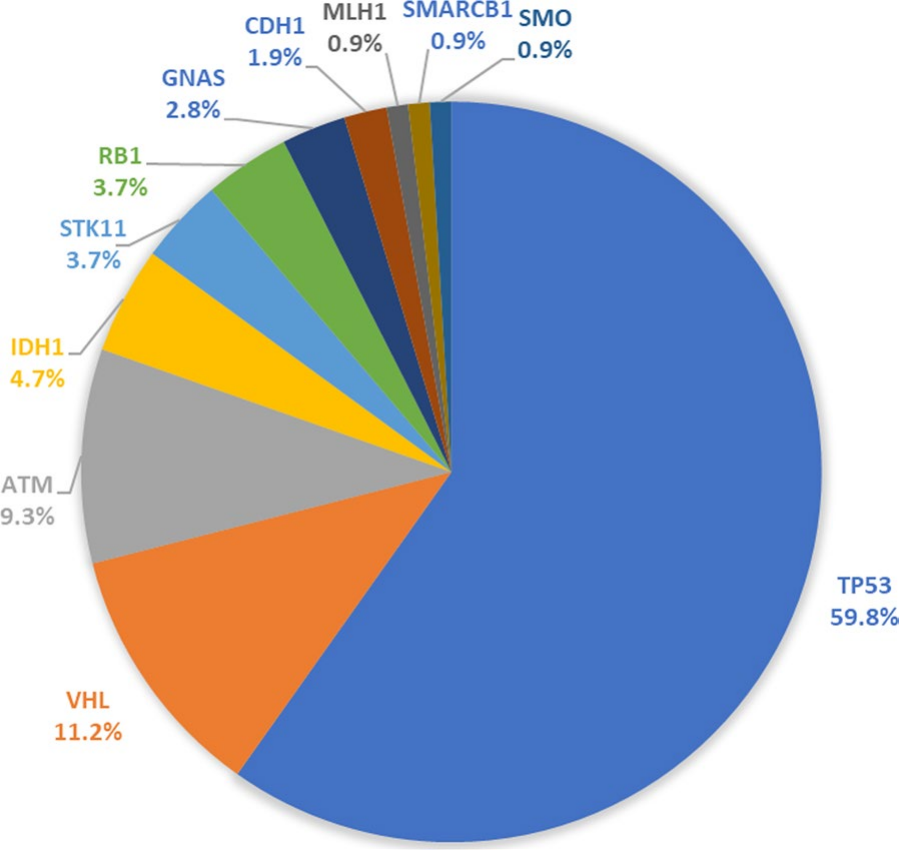
Pie chart showing frequency of genetic variants in HER2-positive breast cancer patients

A total of 107 genetic variants in 11 genes were identified in 19 HER2-positive breast cancer patients. Most of the genetic variants (59.8%) were identified in *TP53* gene, followed by *VHL* (11.2%), and *ATM* (9.3%) (Fig. 1). No mutations were identified in six genes (*NPM1, CDKN2A, EZH2, IDH2, GNA11*, and *GNAQ*)*.* The mutation frequency ranged from 2 to 13 variants per patient with a mean of 5 variants.

**Fig. 2:**
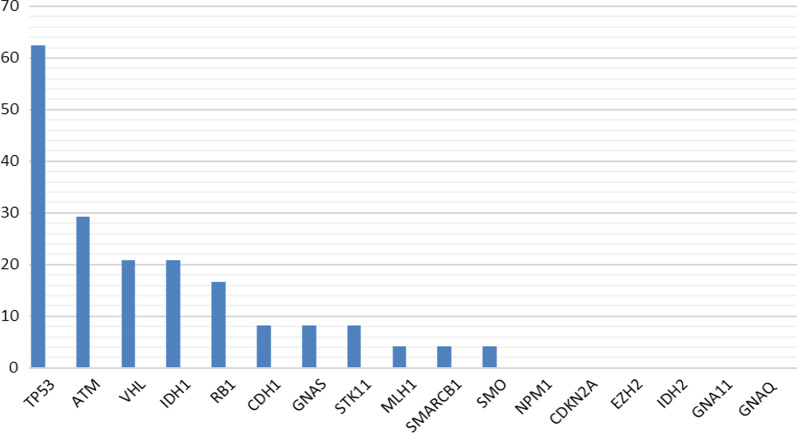
The mutational prevalence of the studied genes in HER2-positive breast cancer patients (*n* = 24)

The mutational prevalence of the studied genes among patients varied widely from 62.5 to 0%. The most frequently mutated gene was *TP53* in 62.5% of the patients, followed by *ATM* (29.2%), *VHL* (20.8%), and *IDH1* (20.8%) (Fig. 2).

**Fig. 3:**
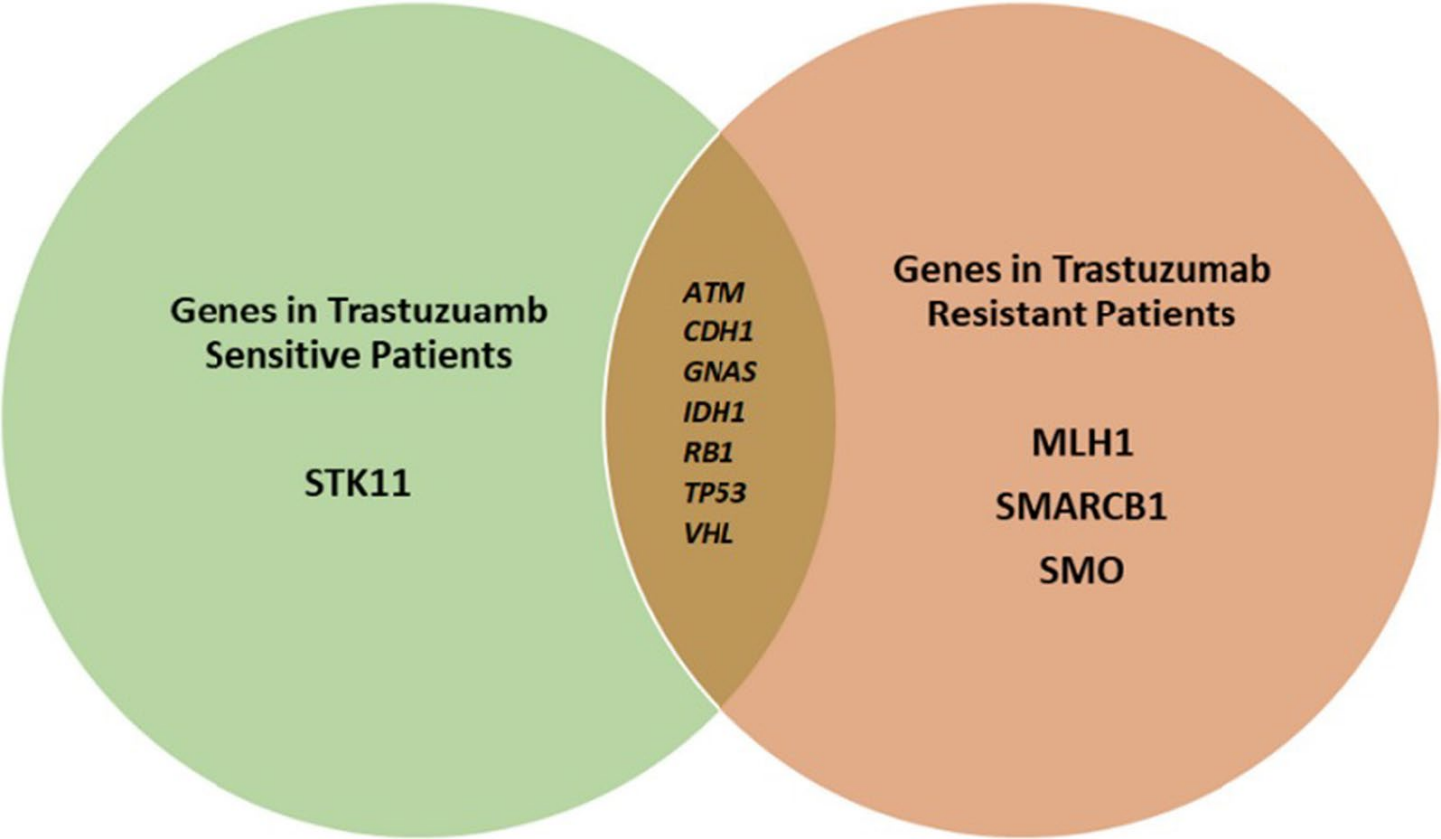
A Venn diagram showing distribution of genes in HER2-positive breast cancer patients

Seven genes were identified to be mutated in both trastuzumab-sensitive and trastuzumab-resistant patients (*ATM*, *CDH1*, *GNAS*, *IDH1, RB1, TP53,* and *VHL*). *STK11* gene was mutated only in the sensitive group. On the other hand, *MLH1, SMARCB1,* and *SMO* genes were only mutated in the resistant group (Fig. 3).

### SNV in trastuzumab-sensitive and trastuzumab-resistant patients

**Fig. 4:**
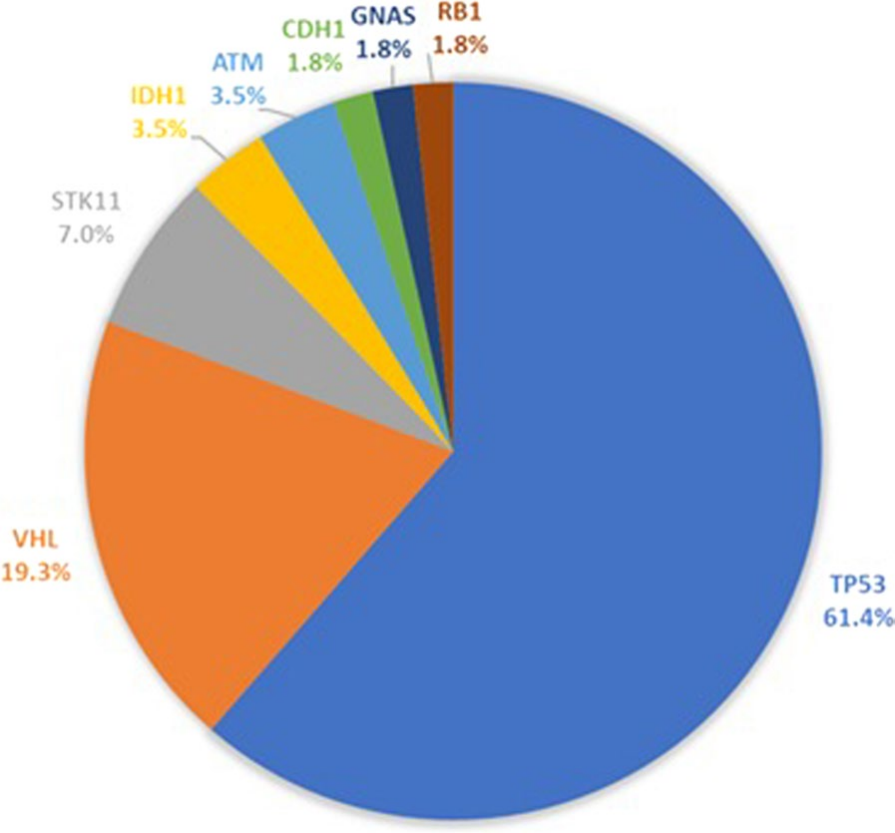
Pie chart showing frequency of genetic variants in trastuzumab-sensitive patients

**Fig. 5:**
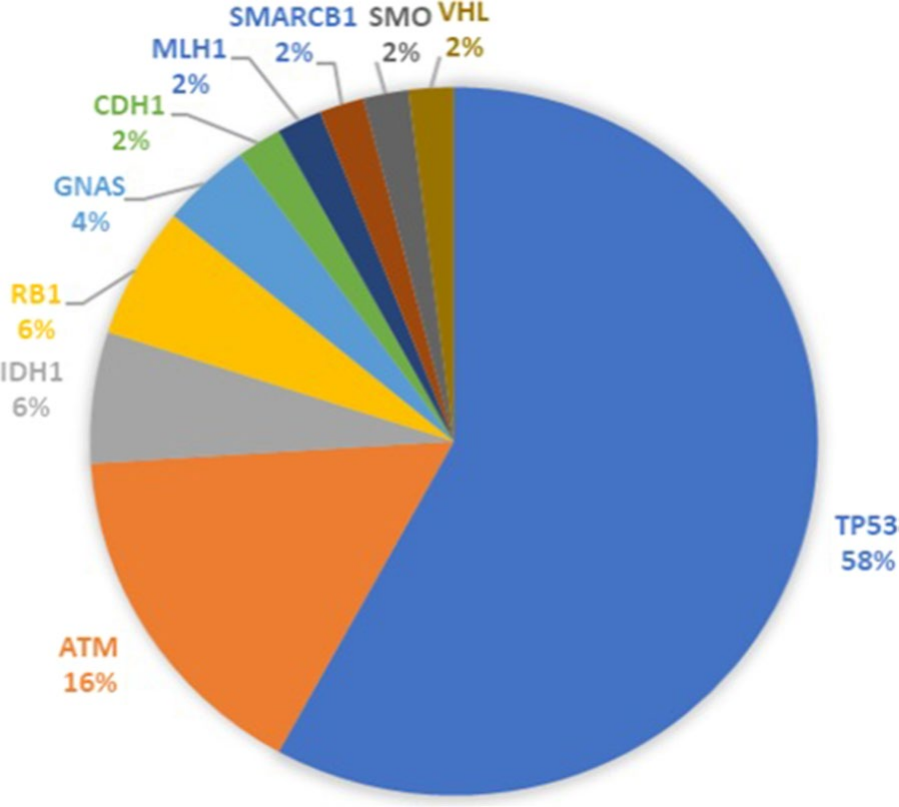
Pie chart showing frequency of genetic variants in trastuzumab-resistant patients

A total of 57 genetic variants were identified in eight genes (*ATM*, *CDH1*, *GNAS*, *IDH1, RB1, STK11, TP53,* and *VHL*) in ten trastuzumab-sensitive patients (83.3%). While, in trastuzumab-resistant group, a total of 50 genetic variants were identified in ten genes (*ATM, CDH1, GNAS, IDH1, MLH1, RB1, SMARCB1, SMO, TP53,* and *VHL*) in nine patients (75%). The most frequently mutated gene was *TP53* in both groups followed by *VHL* in trastuzumab-sensitive patients and *ATM* in trastuzumab-resistant group (Figs. 4, 5) (Additional file 1: Tables S1, S2).

Missense variants were the most common variant type in both sensitive and resistant groups accounting for 63.2% and 76% of variants, respectively. We also found 77.1% of *TP53* variants in the sensitive group were missense variants, followed by nonsense variants (8.6%), synonymous variants (5.7%), frameshift variants (5.7%), and indels (2.9%). *TP53* variants were located mainly within exon 6, exon 7, and exon 4, respectively. In trastuzumab-resistant group, 75.9% of *TP53* variants were missense variants, followed by frameshift variants (10.4%), splice-site variants (6.9%), synonymous variants (3.4%), and indels (3.4%). They were located mainly within exon 6, exon 7, exon 4, and exon 9, respectively (Additional file 1: Tables S3, S4).

**Fig. 6:**
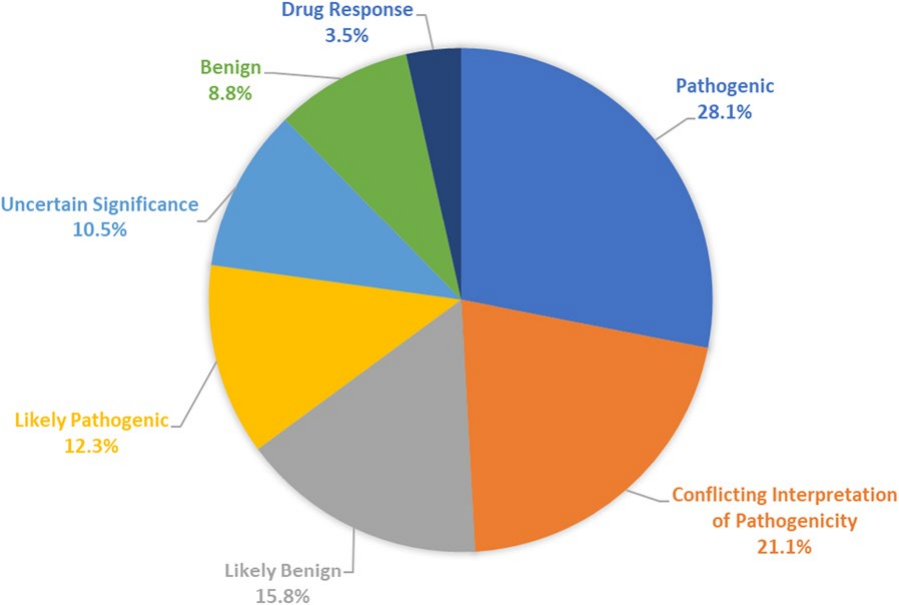
Pie chart showing distribution of variants according to clinical significance in trastuzumab-sensitive patients

**Fig. 7:**
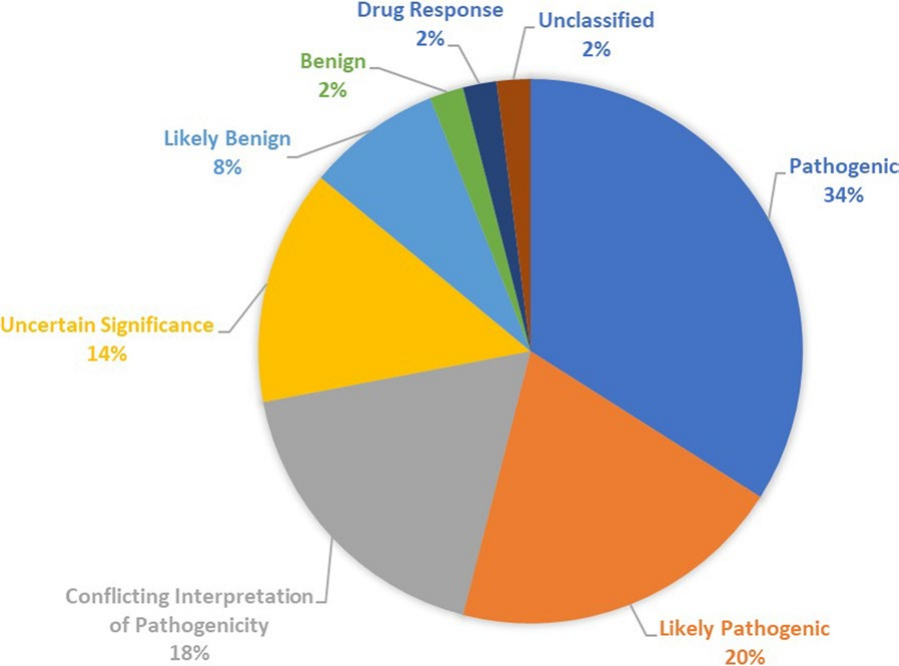
Pie chart showing distribution of variants according to clinical significance in trastuzumab-resistant patients

Regarding clinical significance, pathogenic variants were the most common variants in HER2-positive breast cancer patients accounting for 28.1% of variants in the sensitive group and 34% of variants in the resistant group. Meanwhile, likely pathogenic variants accounted for 12.3% of variants in the sensitive group but 20% of variants in the resistant group (Figs. 6, 7). Similarly, *TP53* pathogenic and likely pathogenic variants together were the most common variants in *TP53* gene in both groups. They accounted together for 34.2% of variants in the sensitive group and almost half of variants (48.3%) in the resistant group (Additional file 1: Tables S5, S6).

### SNV associated with targeted therapy response

By comparing the genetic variants in the sensitive group to the resistant group, we identified 29 variants in nine genes (*ATM, CDH1, GNAS, MLH1, RB1, SMARCB1, SMO, TP53,* and *VHL*) in nine patients that only occurred in trastuzumab-resistant patients and could be associated with resistance to trastuzumab therapy; nine pathogenic variants, five likely pathogenic variants, seven variants with uncertain significance, four likely benign variants, one benign variant, two with conflicting interpretation with pathogenicity, and one novel variant allele (Table 2). Some of these variants was repeated in more than one patient.

**Table 2:** Genetic variants only identified in Trastuzumab-resistant group

Patient no	# Locus	Type	Gene	Transcript	Function	Exon	Protein	Coding	Clinvar	dSNP
No. 1	chr17:7577559	SNV	TP53	NM_000546.6	Missense	6	p.Ser241Phe	c.722C > T	Likely pathogenic	rs28934573
	chr17:7578214	SNV	TP53	NM_000546.6	Synonymous	5	p.Thr211 =	c.633T > G	Likely benign	rs976434163
	chr17:7577035	INDEL	TP53	NM_000546.6	Frameshift	7	p.Gly302ArgfsTer4	c.902_903insC	Pathogenic	rs876660726
	chr17:7578523	SNV	TP53	NM_000546.6	Missense	4	p.Gln136Arg	c.407A > G	–	rs1567554216
	chr11:108205810	SNV	ATM	NM_000051.4	Missense	54	p.Gly2709Ser	c.8125G > A	Uncertain significance	rs3218680
	chr11:108205781	SNV	ATM	NM_000051.4	Missense	54	p.Pro2699Leu	c.8096C > T	Uncertain significance	rs879254209
	chr11:108236213	SNV	ATM	NM_000051.4	Missense	62	p.Pro3050Leu	c.9149C > T	Uncertain significance	rs778267979
	chr11:108170511	SNV	ATM	NM_000051.4	Missense	33	p.Lys1692Asn	c.5076A > C	Uncertain significance	rs767841041
	chr16:68835663	SNV	CDH1	NM_004360.5	Missense	3	p.Val85Ala	c.254T > C	Uncertain significance	rs878854688
No. 2	chr17:7577559	SNV	TP53	NM_000546.6	Missense	6	p.Ser241Phe	c.722C > T	Likely pathogenic	rs28934573
	chr17:7578555	Duplication	TP53	NM_000546.6	Splice acceptor	-	p.?	c.376-2dup	Conflicting Interpretation	rs751253294
	chr13:48955550	SNV	RB1	NM_000321.3	Nonsense	17	p.Arg556Ter	c.1666C >	Pathogenic	rs121913304
No. 3	chr20:57484421	SNV	GNAS	NM_000516.7	Missense	8	p.Arg201His	c.602G > A	Pathogenic	rs121913495
	chr13:48955550	SNV	RB1	NM_000321.3	Nonsense	17	p.Arg556Ter	c.1666C > T	Pathogenic	rs121913304
	chr3:10188263	Delet-ion	VHL	NM_000551.4	Frameshift	2	p.Phe136fs	c.408del	Pathogenic	rs397516442
	chr3:37067240	SNV	MLH1	NM_000249.4	Missense	12	p.Val384Asp	c.1151T > A	Benign	rs63750447
No. 4	chr17:7577124	SNV	TP53	NM_000546.6	Missense	7	p.Val272Met	c.814G > A	Pathogenic	rs121912657
	chr17:7577128	SNV	TP53	NM_000546.6	Missense	7	p.Phe270Leu	c.810 T > G	Likely Pathogenic	rs1057519987
No. 5	chr7:128850341	SNV	SMO	NM_005631.5	Missense	9	p.Trp535Leu	c.1604G > T	Pathogenic	rs121918347
	chr11:108180945	SNV	ATM	NM_000051.4	Missense	38	p.Val1941Leu	c.5821G > C	Conflicting interpretation	rs147187700
No. 6	chr17:7578547	SNV	TP53	NM_000546.6	Missense	4	p.Pro128Leu	c.383C > T	Uncertain Significance	rs1597371657
	chr17:7578553	SNV	TP53	NM_000546.6	Missense	4	p.Tyr126Cys	c.377A > G	Uncertain Significance	rs1555526335
	chr17:7578555	Duplication	TP53	NM_000546.6	Splice acceptor	-	p.?	c.376-2dup	Conflicting Interpretation	rs751253294
No. 7	chr13:49039164	SNV	RB1	NM_000321.3	Nonsense	22	p.Glu748Ter	c.2242G > T	Pathogenic	rs121913297
	chr11:108180945	SNV	ATM	NM_000051.4	Missense	38	p.Val1941Leu	c.5821G > C	Conflicting interpretation	rs147187700
No. 8	chr17:7573996	SNV	TP53	NM_000546.6	Missense	9	p.Leu344Pro	c.1031T > C	Likely Pathogenic	rs121912662
	chr17:7574017	SNV	TP53	NM_000546.6	Missense	9	p.Arg337Pro	c.1010G > C	Likely Pathogenic	rs121912664
	chr17:7574012	SNV	TP53	NM_000546.6	Missense	9	p.Glu339Gln	c.1015G > C	Likely Benign	rs17882252
	chr17:7573931	SNV	TP53	NM_000546.6	Missense	9	p.Ser366Ala	c.1096 T > G	Likely Benign	rs17881470
No.9	chr17:7577521	SNV	TP53	NM_000546.6	Missense	6	p.Ile254Val	c.760A > G	Likely benign	rs746601313
	chr17:7577515	INDEL	TP53	NM_000546.6	Indel	6	p.Ile255del	c.761TCA[1]	Likely Pathogenic	rs1064794309
	chr17:7577522	dele-tion	TP53	NM_000546.6	Frameshift	6	p.Ile254fs	c.759del	Pathogenic	rs1567549129
	chr22:24133967	SNV	SMARCB1	NM_003073.5	Nonsense	2	p.Arg40Ter	c.118C > T	Pathogenic	rs1060503015

Sixteen variants were identified in *TP53* gene (55.2%) in six trastuzumab-resistant patients, these variants were mainly distributed in exons 6, exon 7, and exon 9. Five variants were identified in *ATM* gene (17.2%) in three resistant patients, they were located mainly in exon 38 and exon 54. In addition, two *RB1* variants (6.9%) located in exon 17 and exon 22 were identified in three resistant patients. Four resistant patients were found to harbor the remaining six variants that were identified in *CDH1, GNAS, MLH1, SMARCB1, SMO*, and *VHL* genes (20.7%).

Interestingly, four variants were found to be repeated in more than one resistant patient. Two variants were in *TP53* gene; a likely pathogenic variant p.Ser241Phe in exon 6 that leads to replacement of serine at codon 241 by phenylalanine [20], this variant was identified in two resistant patients (No. 1 and No. 2). Another splice site variant c.376-2dup, which affects mRNA splicing resulting in abnormal protein. This variant was identified in two resistant patients (No. 2 and No. 6). It was reported to have conflicting interpretations of pathogenicity with mainly uncertain significance [21]. The third variant was in *ATM* gene; p.Val1941Leu and was identified in two resistant patients (No. 5 and No. 7). It is located in exon 38 with conflicting interpretations of pathogenicity. It results from a G to C substitution at nucleotide position 5821 which replaces valine by leucine at codon 1941 [22]. The last variant is a pathogenic *RB1* variant p.Arg556Ter located in exon 17. It was identified in two resistant patients (No. 2 and No. 3). It is a nonsense variant that results from replacing C by T nucleotide at position 1666 leading to a premature stop codon at codon 556 [23].

In addition, we identified three variants in *MLH1, SMARCB1, and SMO* genes*.* These genes were only mutated in the resistant group. *MLH1* p.Val384Asp is a missense benign variant located in exon 12, where valine is replaced by aspartic acid at codon 384 [24]. It was identified in resistant patient (No. 3) who was diagnosed with Luminal B (ER+, PR+, HER2+) breast cancer in stage IIIA and grade II. This patient underwent surgery and received Adriamycin, Cyclophosphamide, Taxol, Tamoxifen and Herceptin. However, she developed pulmonary metastasis and died later. *SMARCB1* p.Arg40Ter is a nonsense pathogenic variant within exon 2 which results in a premature translational stop signal at codon 40 of *SMARCB1* gene [25]. It was identified in resistant patient (No. 9). Lastly, *SMO* p.Trp535Leu is also a pathogenic but missense variant located in exon 9 [26] which was identified in resistant patient (No. 5).

Furthermore, a novel allele (c.407A > G, p.Gln136Arg) within exon 4 in *TP53* (rs1567554216) was identified in resistant patient (No. 1). *TP53* p.Gln136Arg is a missense variant where amino acid glutamine is replaced by arginine at codon 136 (Q [CAA] > R [CGA]) of the TP53 protein due to A to G substitution at nucleotide position 407. Resistant patient (No. 1) was diagnosed with HER2-enriched breast cancer (ER−, PR−, HER2+) in stage IIIA and grade II. She was first diagnosed with a right breast mass lesion and right axillary lymph node metastasis to which she underwent radical mastectomy and treatment with Adriamycin/ Cyclophosphamide, Taxotere, and Herceptin. However, 1 year later she developed a local recurrence with skin infiltration. Genetic analysis of this patient identified seven *TP53* variants (p.Ser241Phe, p.Thr211=, p.Cys135Gly, p.Arg248Leu, p.Arg249Ser, p.Gly302ArgfsTer4, and p.Gln136Arg), four *ATM* variants (p.Gly2709Ser, p.Pro2699Leu, p.Pro3050Leu, and p.Lys1692Asn), *CDH1* p.Val85Ala and *IDH1* p.Arg132His.

Resistant patient (No. 2) was diagnosed with HER2-enriched left breast cancer (ER−, PR−, HER2+) in stage III and grade III. She received systematic treatment. Unfortunately, 10 months later, she complained of neurological symptoms (numbness and tingling), MRI Brain with GAD revealed metastatic deposits, and additionally, CT chest revealed pulmonary metastatic nodules. The genetic analysis revealed five *TP53* variants (p.Cys135Gly, p.Ser241Phe, p.Arg249Ser, p.Arg248Leu, and a splice site variant c.376-2dup), *ATM* p.Arg3008Cys, *IDH1* p.Arg132His, and *RB1* p.Arg556Ter. Most identified variants were pathogenic and likely pathogenic (75%) and three variants (*TP53* p.Ser241Phe, *TP53* c.376-2dup and *RB1* c.1666C > T) of which were only repeated in other resistant patients.

## Discussion

HER2-positive breast cancers account 15–20% of all breast cancer cases and show aggressive course and a poor prognosis [2, 3]. Currently, there are many FDA-approved HER2-targeted therapies including monoclonal antibodies (e.g., trastuzumab and pertuzumab), antibody–drug conjugates (e.g., T-DM1 and DS-8201), and small-molecule HER1/2 TKIs (e.g., lapatinib, neratinib, and tucatinib) [27]. Trastuzumab (Herceptin) is the first FDA-approved and is key for treatment of HER2-positive breast cancer [28]. Although trastuzumab significantly improves disease-free survival (DFS), about 25% of patients with early-stage HER2-positive breast cancer disease will relapse after trastuzumab treatment. It may be attributed to the mutation of the target itself after anti-HER2 treatment with down-regulation or loss of HER2 expression, which leads to changes in drug binding or mutations in the HER2 downstream intracellular signaling pathways which when activated, promote tumorigenesis such as PI3K/ AKT/mTOR pathway [27, 29].

In the era of individualized precision medicine, the application of NGS allows the detection of genetic aberrations, which could serve as potential biomarkers for predicting trastuzumab resistance. Consequently, these markers can separate patients who would benefit only from monotherapy from high-risk patients who require combination therapy with adjustment of treatment plans, to ensure favorable prognosis and effectively reduce the treatment cost and side effects [11, 30, 31]. A number of adverse events have been linked to the use of trastuzumab, including acute cardiac toxicity, minor hematologic deficiencies, gastrointestinal symptoms, and pulmonary symptoms [32]. In case of trastuzumab resistance, other drugs can be used such as small molecule TKIs either alone or with monoclonal antibodies, or an ADCs. The second-generation monoclonal antibody margetuximab has been also approved by the FDA for use with chemotherapy for the treatment of previously treated metastatic HER2-positive breast cancer [27].

NGS platform was used in the current study to survey genetic mutations in selected genes that could confer resistance to trastuzumab therapy using FFPE samples. Genetic analysis revealed the presence of 107 genetic variants in HER2-positive breast cancer patients. The most frequent genetic variants were found in *TP53* gene*,* followed by *VHL* and *ATM* genes.

By comparing the genetic variants in the sensitive group to the resistant group, 29 variants were identified in nine genes (*ATM, CDH1, GNAS, MLH1, RB1, SMARCB1, SMO, TP53,* and *VHL*) in nine patients that only occurred in trastuzumab-resistant patients and could be associated with resistance to anti-HER2-targeted therapy. The most frequent variants were identified in *TP53.* Pathogenic and likely pathogenic *TP53* variants were found to be more frequent in the resistant group compared to the sensitive group.

Similarly, Ye et al. studied the response to trastuzumab using 24 cfDNA samples from 20 patients with HER2‑positive metastatic breast cancer. They reported that genetic variants in *TP53* gene were among the most frequent genetic variants in their study [33]. p53 signaling pathway is activated when cells are under stress such as DNA damage. Upon activation, p53 protein works as a transcription factor that transactivates multiple target genes that initiate cell cycle arrest, apoptosis, DNA repair and inhibit metastasis [34, 35]. Breast cancer is actually reported to be the most common cancer (25.5%) in women with pathogenic *TP53* mutations [36, 37]. Moreover, the percentage *TP53* mutations could reach 70% of HER2-positive breast cancer patients both correlating with a poor prognosis [38–40]. *TP53* loss of function mutations were found to be associated with resistance to cytotoxic anticancer drugs in breast cancer patients [41]. However, Fountzilas et al. reported that p53-mutated tumors had longer disease-free survival in patients treated with trastuzumab compared to patients not treated with the drug [42]. Therefore, they suggested that the combination of HER2-targeted drugs with anti-mutp53 therapy could provide a synergistic effect in treatment of HER2-positive breast cancer patients [40].

Interestingly, two variants in *TP53* gene were found to be present in more than one resistant patient; p.Ser241Phe and c.376-2dup. p.Ser241Phe is a likely pathogenic variant located in exon 6, it was identified in two resistant patients. It was previously reported by Chang et al. in breast cancer patients [43]. c.376-2dup is a splice site variant that was previously reported by Hauke et al. in breast cancer [44]. It was found in two resistant patients in our study. However, the implication of these two variants in treatment response is not clear.

We also identified a novel allele (c.407A > G, p.Gln136Arg) within exon 4 in *TP53* variant (rs1567554216) in one resistant patient. The variant rs1567554216 was reported in Li–Fraumeni syndrome but with allele (c.407A > C, p.Gln136Pro) where glutamine is replaced by proline at codon 136. The latter variant *TP53* p.Gln136Pro is considered of uncertain significance [45–47].

Our results showed that *ATM* mutations were more frequent in resistant patients compared to sensitive patients. *ATM* variant p.Val1941Leu in exon 38 was found in two resistant patients. It was reported to cause reduced ATM protein level and kinase activity [48]. *ATM* works as a tumor suppressor gene with a central role in DNA damage response due to double-stranded breaks [14]. *ATM* mutations were found to be associated with an increased risk of breast cancer and *ATM* loss of function was reported in familial breast cancer patients [22, 49, 50]. Stagni et al., reported that ATM protein activity could enhance HER2-dependent tumorigenicity and ATM works as a novel modulator of HER2 protein stability by preventing HER2 degradation. They also reported that *ATM* inhibition or loss of function could induce trastuzumab resistance [14].

Moreover, a pathogenic variant *RB1* p.Arg556Ter in exon 17 was found also in two resistant patients. However, this variant was mainly reported in retinoblastoma [51, 52]. *RB1* is a tumor suppressor gene that is mutated in several types of cancer [53]. *RB1* gene under-expression was actually reported to promote breast carcinogenesis [54]. Moreover, RB pathway is frequently altered in HER2+ tumors [55]. Risi et al. [15] suggested that *RB1* loss of function gene signature (RBsig) could predict response to neoadjuvant chemotherapy in combination with trastuzumab, lapatinib or both in breast cancer.

In addition, we found three genes *MLH1*, *SMARCB1,* and *SMO* that were only mutated in the resistant group. p.Val384Asp in *MLH1* gene is a benign variant, however, Lee et al. [56] reported that this variant has high prevalence in HER2-positive luminal B breast cancer which is correlated with breast cancer molecular subtype in our patient harboring this variant. Chiu et al. [24] also stated that *MLH1* p.Val384Asp is associated with poor response to EGFR tyrosine kinase inhibitors but in patients with lung adenocarcinoma. Qing Ye et al. reported *MLH1* as Herceptin resistance-associated gene. They identified four *MLH1* variants (p.Phe155Ser, p.Gln168Lys, p.Val143Asp, and p.Ser160Asn) only present in Herceptin-resistant HER2-positive breast cancer patients [33]. The activity of the mismatch repair system is crucial for removal of several polymerase errors, including base substitution and insertion-deletion mismatches that can form during the replication [57]. Loss of function of *MLH1* gene was reported to be associated with resistance to anticancer drugs and poor disease-free survival [58].p.Arg40Ter variant in *SMARCB1*gene is a pathogenic variant that was reported to have a predisposition to various cancers but mainly rhabdoid tumors [25]. *SMO* p.Trp535Leu is a missense pathogenic variant, it was reported by Xie et al. [26] but in basal cell carcinoma. *SMO* gene is one of the genes in Hedgehog (HH) signaling pathway whose expression correlates with tumor size, metastasis, and recurrence. Thus, it can be targeted by SMO inhibitors which are investigated for treatment of breast, liver, and colon cancer. However, *SMO* mutations can cause resistance to these inhibitors [59, 60].p.Arg201His variant was detected in *GNAS* gene in one resistant patient*.* It is a pathogenic variant that was previously reported in breast cancer [43]. *GNAS* gene was one of the anti‑HER2 therapy resistance‑associated genes reported by Qing et al. who detected seven *GNAS* variants (p.Arg186His, p.Asp181Gly, p.Asn203Ser, p.Arg216Leu, p.Met206Val, p.Arg216Cys, and p.Asp214Asn) only present in Herceptin-resistant breast cancer patients [33]. GNAS was found to induce breast cancer cell proliferation and metastasis through the PI3K/AKT/Snail1/E-cadherin signaling pathway [61].

We also identified some other variants (*TP53* p.Pro3050Leu, *TP53* p.Val272Met, *TP53* p.Phe270Leu, *TP53* p.Arg337Pro, and *TP53* p.Ile254Va) in the resistant group that have been previously reported in breast cancer [43, 62, 63]. However no association with treatment was found.

Moreover, 11 common genetic variants were identified in four genes (*TP53, IDH1, ATM,* and *GNAS)* in both sensitive and resistant groups. Eight variants were in *TP53* gene (p.Cys135Gly, p.Glu349fs, p.Arg248Leu, p.Pro278Ser, p.Asp281Ala, p.Arg249Ser, p.Ser127Phe, and p.Arg280Gly), three variants in *IDH1* gene (p.Arg132His), *ATM* gene (p.Arg3008Cys), and *GNAS* gene (p.Arg201Cys). Interestingly, Chang et al. [43] reported the same seven variants (*TP53* p.Cys135Gly, *TP53* p.Arg248Leu, *TP53* p.Pro278Ser, *TP53* p.Asp281Ala, *TP53* p.Arg280Gly, *IDH1* p.Arg132His, and *GNAS* p.Arg201Cys) in breast neoplasm in their study. The similarity of results could highlight the pathogenic contribution of these variants in breast cancer specifically *TP53* variants that cause dysregulated p53 signaling pathway which is an early incident in breast tumorigenesis [35]. In addition, *TP53* variant p.Glu349fs was reported to be associated with response to PARP inhibitors but in prostate cancer [64].

The main limitations of this study were the small number of cases included, and lack of study of the underlying molecular mechanisms by which the detected variants can cause trastuzumab resistance. Therefore, we recommended future studies with a larger sample size to confirm the association between the detected genetic variants and trastuzumab response and to study the mechanisms by which the detected variants could affect trastuzumab response in order to discover new therapeutic targets. In addition, we recommend to study the association between genetic variants and other anti-HER2 drugs.

Based on previous findings, we concluded that targeted next-generation sequencing is a useful tool to detect DNA mutations that could have clinical utility in predicting response to anti-HER2-targeted therapy allowing individualized treatment regimens for HER2-positive breast cancer patients.

## Supplementary Information

**Additional file 1:** Supplementary tables.

## Data Availability

The data supporting the conclusions are included within the article.
