# Supplementary material for: Genetic mutations in HER2-positive breast cancer: possible association with response to trastuzumab therapy
Source: Hum Genomics. 2023 May 18;17:43. doi: 10.1186/s40246-023-00493-5 (PMC10193616; doi:10.1186/s40246-023-00493-5)
Supplement: Supplementary file 1 — Additional file 1: Supplementary tables. [file 40246_2023_493_MOESM1_ESM.docx]

**Supplementary data:**

**Table (S1): The genetic variants identified in Trastuzumab Sensitive Patients**

| **Patient No.** | **# Locus** | **Type** | **Gene** | **Transcript** | **Function** | **Exon** | **Protein** | **Coding** | **Clinvar** | **dSNP** | **AF%** |
| --- | --- | --- | --- | --- | --- | --- | --- | --- | --- | --- | --- |
| No. 1 | chr17: 7578393 | SNV | TP53 | NM_000546.6 | missense | 4 | p.His179Gln | c.537T>A | Pathogenic | rs876660821 | 5.14 |
|  | chr17:7578212 | SNV | TP53 | NM_000546.6 | nonsense | 5 | p.Arg213Ter | c.637C>T | Pathogenic | rs397516436 | 2.02 |
|  | chr17:7579472 | SNV | TP53 | NM_000546.6 | missense | 3 | p.Pro72Arg | c.215C>G | Benign | rs1042522 | 3.2 |
|  | chr3:10183817 | SNV | VHL | NM_000551.4 | nonsense | 1 | p.Gln96Ter | c.286C>T | Pathogenic | rs1131690959 | 1.55 |
|  | chr3:10183832 | SNV | VHL | NM_000551.4 | synonym-ous | 1 | p.Leu101= | c.301C>T | likely benign | rs772300829 | 1.2 |
|  | chr3:10183772 | SNV | VHL | NM_000551.4 | missense | 1 | p.Pro81Ser | c.241C>T | Conflicting interpretation | rs104893829 | 3.73 |
|  | chr19:1207021 | SNV | STK11 | NM_000455.5 | nonsense | 1 | p.Gln37Ter | c.109C>T | Likely-Pathogenic | rs121913324 | 2.66 |
|  | chr19:1220513 | SNV | STK11 | NM_000455.5 | intronic |  | p.? | c.597+9G>A | likely benign | rs863224361 | 8.33 |
|  | chr19:1221293 | SNV | STK11 | NM_000455.5 | synonym-ous | 6 | p.Tyr272= | c.816C>T | Benign | rs9282859 | 5.35 |
|  | chr11:108236086 | SNV | ATM | NM_000051.4 | missense | 62 | p.Arg3008Cys | c.9022C>T | Pathogenic | rs587782292 | 1.69 |
| No. 2 | chr17:7578413 | SNV | TP53 | NM_000546.6 | missense | 4 | p.Val173Met | c.517G>A | Pathogenic | rs876660754 | 1.55 |
|  | chr17:7579472 | SNV | TP53 | NM_000546.6 | missense | 3 | p.Pro72Arg | c.215C>G | Benign | rs1042522 | 3.44 |
|  | chr17:7577061 | SNV | TP53 | NM_000546.6 | missense | 7 | p.Gly293Arg | c.877G>A | Uncertain significance | rs587780076 | 3.71 |
|  | chr17:7578525 | SNV | TP53 | NM_000546.6 | synonym-ous | 4 | p.Cys135= | c.405C>T | Likely benign​ | rs1057519976 | 5.2 |
|  | chr3:10191488 | SNV | VHL | NM_000551.4 | nonsense | 3 | p.Arg161Ter | c.481C>T | Pathogenic | rs5030818 | 2.25 |
|  | chr3:10183832 | SNV | VHL | NM_000551.4 | synonym-ous | 1 | p.Leu101= | c.301C>T | likely benign | rs772300829 | 1.12 |
|  | chr20:57484420 | SNV | GNAS | NM_000516.7 | missense | 8 | p.Arg201Cys | c.601C>T | Likely-Pathogenic | rs11554273 | 2.43 |
|  | chr11:108155132 | SNV | ATM | NM_000051.4 | missense | 26 | p.Ala1309Thr | c.3925G>A | Benign | rs149711770 | 2.8 |
|  | chr16:68847249 | SNV | CDH1 | NM_004360.5 | missense | 9 | p.Val391Ile | c.1171G>A | Uncertain-Significance | rs556110297 | 3.55 |
|  | chr13:48942685 | SNV | RB1 | NM_000321.3 | nonsense | 11 | p.Arg358Ter | c.1072C>T | Pathogenic | rs121913301 | 5.14 |
|  | chr19:1220513 | SNV | STK11 | NM_000455.5 | intronic |  | p.? | c.597+9G>A | likely benign | rs863224361 | 3.14 |
| No. 3 | chr17:7579472 | SNV | TP53 | NM_000546.6 | missense | 3 | p.Pro72Arg | c.215C>G | Benign | rs1042522 | 6.1 |
|  | chr17:7573982 | Delet-ion | TP53 | NM_000546.6 | Frameshift | 9 | p.Glu349fs | c.1045del | Drug-Response | rs1057519625 | 3.02 |
|  | chr17:7577538 | SNV | TP53 | NM_000546.6 | missense | 6 | p.Arg248Leu | c.743G>T | Pathogenic | rs11540652 | 5.4 |
|  | chr17:7577106 | SNV | TP53 | NM_000546.6 | missense | 7 | p.Pro278Ser | c.832C>T | Conflicting interpretation | rs17849781 | 2.1 |
|  | chr17:7577534 | SNV | TP53 | NM_000546.6 | missense | 6 | p.Arg249Ser | c.747G>T | Conflicting interpretation | rs28934571 | 1.12 |
|  | chr17:7578263 | SNV | TP53 | NM_000546.6 | nonsense | 5 | p.Arg196Ter | c.586C>T | Pathogenic | rs397516435 | 2.2 |
| No. 4 | chr3:10183772 | SNV | VHL | NM_000551.4 | missense | 1 | p.Pro81Ser | c.241C>T | Conflicting interpretation | rs104893829 | 5.93 |
|  | chr3:10183787 | SNV | VHL | NM_000551.4 | missense | 1 | p.Pro86Ser | c.256C>T | Likely-Pathogenic | rs398123481 | 3.57 |
|  | chr3:10183794 | SNV | VHL | NM_000551.4 | nonsense | 1 | p.Trp88Ter | c.263G>A | Pathogenic | rs119103277 | 1.75 |
|  | chr3:10183817 | SNV | VHL | NM_000551.4 | nonsense | 1 | p.Gln96Ter | c.286C>T | Pathogenic | rs1131690959 | 3.15 |
|  | chr3:10183832 | SNV | VHL | NM_000551.4 | synonym-ous | 1 | p.Leu101= | c.301C>T | likely benign | rs772300829 | 9.14 |
| No. 5 | chr17:7573982 | Delet-ion | TP53 | NM_000546.6 | frameshift | 9 | p.Glu349fs | c.1045del | Drug-Response | rs1057519625 | 3.36 |
|  | chr17:7577096 | SNV | TP53 | NM_000546.6 | missense | 7 | p.Asp281Ala | c.842A>C | Likely-Pathogenic | rs587781525 | 4.46 |
|  | chr17:7577100 | SNV | TP53 | NM_000546.6 | missense | 7 | p.Arg280Gly | c.838A>G | Conflicting interpretation | rs753660142 | 1.35 |
|  | chr17:7577106 | SNV | TP53 | NM_000546.6 | missense | 7 | p.Pro278Ser | c.832C>T | Conflicting interpretation | rs17849781 | 1.76 |
|  | chr17:7577130 | SNV | TP53 | NM_000546.6 | missense | 7 | p.Phe270Ile | c.808T>A | Likely-Pathogenic | rs1057519988 | 2.89 |
| No. 6 | chr17:7577565 | SNV | TP53 | NM_000546.6 | missense | 6 | p.Asn239Ser | c.716A>G | Likely Pathogenic | rs1057519999 | 5.2 |
|  | chr17:7577538 | SNV | TP53 | NM_000546.6 | missense | 6 | p.Arg248Leu | c.743G>T | Pathogenic | rs11540652 | 2.35 |
|  | chr17:7577526 | SNV | TP53 | NM_000546.6 | missense | 6 | p.Leu252Pro | c.755T>C | Conflicting interpretation | rs121912653 | 1.35 |
|  | chr17:7577534 | SNV | TP53 | NM_000546.6 | missense | 6 | p.Arg249Ser | c.747G>T | Conflicting-Interpretation | rs28934571 | 2.71 |
|  | chr17:7577575 | SNV | TP53 | NM_000546.6 | missense | 6 | p.Tyr236Asp | c.706T>G | Conflicting-Interpretation | rs587782289 | 5.16 |
|  | chr2:209113112 | SNV | IDH1 | NM_005896.4 | missense | 4 | p.Arg132His | c.395G>A | Pathogenic | rs121913500 | 2.63 |
| No. 7 | chr17:7578527 | SNV | TP53 | NM_000546.6 | missense | 4 | p.Cys135Gly | c.403T>G | Likely Pathogenic | rs1057519975 | 2.35 |
|  | chr17:7578525 | SNV | TP53 | NM_000546.6 | synonym-ous | 4 | p.Cys135= | c.405C>T | Likely Benign | rs1057519976 | 4.09 |
|  | chr17:7578548 | SNV | TP53 | NM_000546.6 | missense | 4 | p.Pro128Thr | c.382C>A | Uncertain Significance | rs1555526327 | 6.98 |
|  | chr17:7578550 | SNV | TP53 | NM_000546.6 | missense | 4 | p.Ser127Phe | c.380C>T | Conflicting interpretations | rs730881999 | 1.45 |
| No. 8 | chr17:7579365 | SNV | TP53 | NM_000546.6 | missense | 3 | p.Gly108Ser | c.322G>A | Likely benign​ | rs587782461 | 5.7 |
|  | chr17:7579391 | SNV | TP53 | NM_000546.6 | missense | 3 | p.Ser99Phe | c.296C>T | Uncertain Significance | rs1567556019 | 1.91 |
|  | chr17:7579400 | INDEL | TP53 | NM_000546.6 | Indel | 3 | p.Ser96del | c.283TCT[1] | Uncertain significance | rs878854068 | 3.57 |
| No.9 | chr17:7577022 | SNV | TP53 | NM_000546.6 | nonsense | 7 | p.Arg306Ter | c.916C>T | Pathogenic | rs121913344 | 3.1 |
|  | chr17:7577028 | SNV | TP53 | NM_000546.5 | missense | 7 | p.Thr304Ala | c.910A>G | Uncertain significance | rs587782654 | 5.12 |
| No. 10 | chr17:7577526 | SNV | TP53 | NM_000546.6 | missense | 6 | p.Leu252Pro | c.755T>C | Conflicting interpretation | rs121912653 | 5.03 |
|  | chr17:7577534 | SNV | TP53 | NM_000546.6 | missense | 6 | p.Arg249Ser | c.747G>T | Conflicting Interpretation | rs28934571 | 3.76 |
|  | chr17:7577538 | SNV | TP53 | NM_000546.6 | missense | 6 | p.Arg248Leu | c.743G>T | Pathogenic | rs11540652 | 3.64 |
|  | chr2:209113112 | SNV | IDH1 | NM_005896.4 | missense | 4 | p.Arg132His | c.395G>A | Pathogenic | rs121913500 | 2.2 |
|  | chr3:10183832 | SNV | VHL | NM_000551.4 | synonym-ous | 1 | p.Leu101= | c.301C>T | likely benign | rs772300829 | 1.35 |

**Table (S2): The genetic variants identified in Trastuzumab resistant Patients**

| **Patient No.** | **# Locus** | **Type** | **Gene** | **Transcript** | **Function** | **Exon** | **Protein** | **Coding** | **Clinvar** | **dSNP** | **AF%** |
| --- | --- | --- | --- | --- | --- | --- | --- | --- | --- | --- | --- |
| No. 1 | chr17:7577559 | SNV | TP53 | NM_000546.6 | missense | 6 | p.Ser241Phe | c.722C>T | Likely pathogenic | rs28934573 | 1.3 |
|  | chr17:7578214 | SNV | TP53 | NM_000546.6 | synonym-ous | 5 | p.Thr211= | c.633T>G | likely benign | rs976434163 | 4.66 |
|  | chr17:7578527 | SNV | TP53 | NM_000546.6 | missense | 4 | p.Cys135Gly | c.403T>G | Likely-Pathogenic | rs1057519975 | 1.01 |
|  | chr17:7577538 | SNV | TP53 | NM_000546.6 | missense | 6 | p.Arg248Leu | c.743G>T | Pathogenic | rs11540652 | 3.28 |
|  | chr17:7577534 | SNV | TP53 | NM_000546.6 | missense | 6 | p.Arg249Ser | c.747G>T | Conflicting Interpretation | rs28934571 | 1.1 |
|  | chr17:7577035 | INDEL | TP53 | NM_000546.6 | frameshift | 7 | p.Gly302ArgfsTer4 | c.902_903insC | Pathogenic | rs876660726 | 7.14 |
|  | chr17:7578523 | SNV | TP53 | NM_000546.6 | missense | 4 | p.Gln136Arg | c.407A>G | - | rs1567554216 | 5.45 |
|  | chr11:108205810 | SNV | ATM | NM_000051.4 | missense | 54 | p.Gly2709Ser | c.8125G>A | Uncertain significance | rs3218680 | 9.58 |
|  | chr11:108205781 | SNV | ATM | NM_000051.4 | missense | 54 | p.Pro2699Leu | c.8096C>T | Uncertain significance | rs879254209 | 4.36 |
|  | chr11:108236213 | SNV | ATM | NM_000051.4 | missense | 62 | p.Pro3050Leu | c.9149C>T | Uncertain significance | rs778267979 | 11.6 |
|  | chr11:108170511 | SNV | ATM | NM_000051.4 | missense | 33 | p.Lys1692Asn | c.5076A>C | Uncertain significance | rs767841041 | 5.76 |
|  | chr16:68835663 | SNV | CDH1 | NM_004360.5 | missense | 3 | p.Val85Ala | c.254T>C | Uncertain significance | rs878854688 | 5.93 |
|  | chr2:209113112 | SNV | IDH1 | NM_005896.4 | missense | 4 | p.Arg132His | c.395G>A | Pathogenic | rs121913500 | 1.15 |
| No. 2 | chr17:7578527 | SNV | TP53 | NM_000546.6 | missense | 4 | p.Cys135Gly | c.403T>G | Likely Pathogenic | rs1057519975 | 7.04 |
|  | chr17:7577559 | SNV | TP53 | NM_000546.6 | missense | 6 | p.Ser241Phe | c.722C>T | Likely pathogenic | rs28934573 | 3.96 |
|  | chr17:7577534 | SNV | TP53 | NM_000546.6 | missense | 6 | p.Arg249Ser | c.747G>T | Conflicting interpretation | rs28934571 | 1.8 |
|  | chr17:7577538 | SNV | TP53 | NM_000546.6 | missense | 6 | p.Arg248Leu | c.743G>T | Pathogenic | rs11540652 | 2.5 |
|  | chr17:7578555 | Dupli-cation | TP53 | NM_000546.6 | splice acceptor |  | p.? | c.376-2dup | Conflicting Interpretation | rs751253294 | 4.6 |
|  | chr11:108236086 | SNV | ATM | NM_000051.4 | missense | 62 | p.Arg3008Cys | c.9022C>T | Pathogenic | rs587782292 | 5.9 |
|  | chr2:209113112 | SNV | IDH1 | NM_005896.4 | missense | 4 | p.Arg132His | c.395G>A | Pathogenic | rs121913500 | 3.8 |
|  | chr13:48955550 | SNV | RB1 | NM_000321.3 | nonsense | 17 | p.Arg556Ter | c.1666C> | Pathogenic | rs121913304 | 4.8 |
| No .3 | chr20:57484421 | SNV | GNAS | NM_000516.7 | missense | 8 | p.Arg201His | c.602G>A | Pathogenic​ | rs121913495 | 4.18 |
|  | chr20:57484420 | SNV | GNAS | NM_000516.7 | missense | 8 | p.Arg201Cys | c.601C>T | Likely Pathogenic | rs11554273 | 3.86 |
|  | chr11:108236086 | SNV | ATM | NM_000051.4 | missense | 62 | p.Arg3008Cys | c.9022C>T | Pathogenic | rs587782292 | 1.7 |
|  | chr13:48955550 | SNV | RB1 | NM_000321.3 | nonsense | 17 | p.Arg556Ter | c.1666C>T | Pathogenic | rs121913304 | 3.1 |
|  | chr3:10188263 | Delet-ion | VHL | NM_000551.4 | frameshift | 2 | p.Phe136fs | c.408del | Pathogenic | rs397516442 | 5.04 |
|  | chr3:37067240 | SNV | MLH1 | NM_000249.4 | missense | 12 | p.Val384Asp | c.1151T>A | benign | rs63750447 | 1.28 |
|  | chr2:209113112 | SNV | IDH1 | NM_005896.4 | missense | 4 | p.Arg132His | c.395G>A | Pathogenic | rs121913500 | 2.5 |
| No. 4 | chr17:7577100 | SNV | TP53 | NM_000546.6 | missense | 7 | p.Arg280Gly | c.838A>G | Conflicting interpretation | rs753660142 | 1.51 |
|  | chr17:7577096 | SNV | TP53 | NM_000546.6 | missense | 7 | p.Asp281Ala | c.842A>C | Likely-Pathogenic | rs587781525 | 2.91 |
|  | chr17:7577106 | SNV | TP53 | NM_000546.6 | missense | 7 | p.Pro278Ser | c.832C>T | Conflicting interpretation | rs17849781 | 4.6 |
|  | chr17:7577124 | SNV | TP53 | NM_000546.6 | missense | 7 | p.Val272Met | c.814G>A | Pathogenic | rs121912657 | 5.92 |
|  | chr17:7577128 | SNV | TP53 | NM_000546.6 | missense | 7 | p.Phe270Leu | c.810T>G | Likely Pathogenic | rs1057519987 | 2.43 |
| No. 5 | chr7:128850341 | SNV | SMO | NM_005631.5 | missense | 9 | p.Trp535Leu | c.1604G>T | Pathogenic | rs121918347 | 1.18 |
|  | chr11:108180945 | SNV | ATM | NM_000051.4 | missense | 38 | p.Val1941Leu | c.5821G>C | Conflicting interpretation | rs147187700 | 3.66 |
| No. 6 | chr17:7578550 | SNV | TP53 | NM_000546.6 | missense | 4 | p.Ser127Phe | c.380C>T | Conflicting interpretations | rs730881999 | 4.21 |
|  | chr17:7578547 | SNV | TP53 | NM_000546.6 | missense | 4 | p.Pro128Leu | c.383C>T | Uncertain Significance | rs1597371657 | 1.78 |
|  | chr17:7578553 | SNV | TP53 | NM_000546.6 | missense | 4 | p.Tyr126Cys | c.377A>G | Uncertain Significance | rs1555526335 | 2.12 |
|  | chr17:7578555 | Dupli-cation | TP53 | NM_000546.6 | splice acceptor |  | p.? | c.376-2dup | Conflicting Interpretation | rs751253294 | 3.3 |
| No. 7 | chr13:49039164 | SNV | RB1 | NM_000321.3 | nonsense | 22 | p.Glu748Ter | c.2242G>T | Pathogenic | rs121913297 | 1.64 |
|  | chr11:108180945 | SNV | ATM | NM_000051.4 | missense | 38 | p.Val1941Leu | c.5821G>C | Conflicting interpretation | rs147187700 | 2.7 |
| No. 8 | chr17:7573996 | SNV | TP53 | NM_000546.6 | missense | 9 | p.Leu344Pro | c.1031T>C | Likely Pathogenic | rs121912662 | 5.46 |
|  | chr17:7574017 | SNV | TP53 | NM_000546.6 | missense | 9 | p.Arg337Pro | c.1010G>C | Likely Pathogenic | rs121912664 | 6.34 |
|  | chr17:7574012 | SNV | TP53 | NM_000546.6 | missense | 9 | p.Glu339Gln | c.1015G>C | Likely Benign | rs17882252 | 1.82 |
|  | chr17:7573931 | SNV | TP53 | NM_000546.6 | missense | 9 | p.Ser366Ala | c.1096T>G | Likely Benign | rs17881470 | 4.2 |
|  | chr17:7573982 | Delet-ion | TP53 | NM_000546.6 | frameshift | 9 | p.Glu349fs | c.1045del | Drug Response | rs1057519625 | 2.71 |
| No. 9 | chr17:7577521 | SNV | TP53 | NM_000546.6 | missense | 6 | p.Ile254Val | c.760A>G | Likely benign | rs746601313 | 5.03 |
|  | chr17:7577515 | INDEL | TP53 | NM_000546.6 | Indel | 6 | p.Ile255del | c.761TCA[1] | Likely Pathogenic | rs1064794309 | 4.17 |
|  | chr17:7577522 | dele-tion | TP53 | NM_000546.6 | frameshift | 6 | p.Ile254fs | c.759del | Pathogenic​ | rs1567549129 | 4.6 |
|  | chr22:24133967 | SNV | SMARCB1 | NM_003073.5 | nonsense | 2 | p.Arg40Ter | c.118C>T | Pathogenic | rs1060503015 | 1.93 |

**Table (S3): Distribution of genetic variants according to type in Trastuzumab sensitive patients**

| **Type of Variant** | **TP53**  **(n = 35)** | | **VHL**  **(n = 11)** | | **STK11**  **(n = 4)** | | **IDH1**  **(n = 2)** | | **ATM**  **(n = 2)** | | **CDH1**  **(n = 1)** | | **GNAS**  **(n = 1)** | | **RB1**  **(n = 1)** | | **Total**  **(n = 57)** |
| --- | --- | --- | --- | --- | --- | --- | --- | --- | --- | --- | --- | --- | --- | --- | --- | --- | --- |
|  | **No.** | **%** | **No.** | **%** | **No.** | **%** | **No.** | **%** | **No.** | **%** | **No.** | **%** | **No.** | **%** | **No.** | **%** |  |
| Missense | 27 | 77.1 | 3 | 27.2 | 0 | 0.0 | 2 | 100.0 | 2 | 100.0 | 1 | 100.0 | 1 | 100.0 | 0 | 0.0 | 36 |
| Nonsense | 3 | 8.6 | 4 | 36.4 | 1 | 25.0 | 0 | 0.0 | 0 | 0.0 | 0 | 0.0 | 0 | 0.0 | 1 | 100.0 | 9 |
| Synonymous | 2 | 5.7 | 4 | 36.4 | 1 | 25.0 | 0 | 0.0 | 0 | 0.0 | 0 | 0.0 | 0 | 0.0 | 0 | 0.0 | 7 |
| Frameshift | 2 | 5.7 | 0 | 0.0 | 0 | 0.0 | 0 | 0.0 | 0 | 0.0 | 0 | 0.0 | 0 | 0.0 | 0 | 0.0 | 2 |
| Intronic | 0 | 0.0 | 0 | 0.0 | 2 | 50.0 | 0 | 0.0 | 0 | 0.0 | 0 | 0.0 | 0 | 0.0 | 0 | 0.0 | 2 |
| Indel | 1 | 2.9 | 0 | 0.0 | 0 | 0.0 | 0 | 0.0 | 0 | 0.0 | 0 | 0.0 | 0 | 0.0 | 0 | 0.0 | 1 |

**Table (S4): Distribution of genetic variants according to type in Trastuzumab resistant patients**

| **Type of Variant** | **TP53**  **(n = 29)** | | **ATM**  **(n = 8)** | | **IDH1**  **(n = 3)** | | **RB1**  **(n = 3)** | | **GNAS**  **(n = 2)** | | **CDH1**  **(n = 1)** | | **MLH1**  **(n = 1)** | | **SMARCB1**  **(n = 1)** | | **SMO**  **(n = 1)** | | **VHL**  **(n = 1)** | | **Total**  **(n = 50)** |
| --- | --- | --- | --- | --- | --- | --- | --- | --- | --- | --- | --- | --- | --- | --- | --- | --- | --- | --- | --- | --- | --- |
|  | **No.** | **%** | **No.** | **%** | **No.** | **%** | **No.** | **%** | **No.** | **%** | **No.** | **%** | **No.** | **%** | **No.** | **%** | **No.** | **%** | **No.** | **%** |  |
| Missense | 22 | 75.9 | 8 | 100.0 | 3 | 100.0 | 0 | 0.0 | 2 | 100.0 | 1 | 100.0 | 1 | 100.0 | 0 | 0.0 | 1 | 100.0 | 0 | 0.0 | 38 |
| Nonsense | 0 | 0.0 | 0 | 0.0 | 0 | 0.0 | 3 | 100.0 | 0 | 0.0 | 0 | 0.0 | 0 | 0.0 | 1 | 100.0 | 0 | 0.0 | 0 | 0.0 | 4 |
| Synonymous | 1 | 3.4 | 0 | 0.0 | 0 | 0.0 | 0 | 0.0 | 0 | 0.0 | 0 | 0.0 | 0 | 0.0 | 0 | 0.0 | 0 | 0.0 | 0 | 0.0 | 1 |
| Frameshift | 3 | 10.4 | 0 | 0.0 | 0 | 0.0 | 0 | 0.0 | 0 | 0.0 | 0 | 0.0 | 0 | 0.0 | 0 | 0.0 | 0 | 0.0 | 1 | 100.0 | 4 |
| Splice-site | 2 | 6.9 | 0 | 0.0 | 0 | 0.0 | 0 | 0.0 | 0 | 0.0 | 0 | 0.0 | 0 | 0.0 | 0 | 0.0 | 0 | 0.0 | 0 | 0.0 | 2 |
| Indel | 1 | 3.4 | 0 | 0.0 | 0 | 0.0 | 0 | 0.0 | 0 | 0.0 | 0 | 0.0 | 0 | 0.0 | 0 | 0.0 | 0 | 0.0 | 0 | 0.0 | 1 |

**Table (S5): Distribution of genetic variants according to clinical significance in Trastuzumab sensitive patients**

| **Clinical significance** | **TP53**  **(n = 35)** | | **VHL**  **(n = 11)** | | **STK11**  **(n = 4)** | | **IDH1**  **(n = 2)** | | **ATM**  **(n = 2)** | | **CDH1**  **(n = 1)** | | **GNAS**  **(n = 1)** | | **RB1**  **(n = 1)** | | **Total**  **(n = 57)** |
| --- | --- | --- | --- | --- | --- | --- | --- | --- | --- | --- | --- | --- | --- | --- | --- | --- | --- |
|  | **No.** | **%** | **No.** | **%** | **No.** | **%** | **No.** | **%** | **No.** | **%** | **No.** | **%** | **No.** | **%** | **No.** | **%** |  |
| Benign | 3 | 8.6 | 0 | 0.0 | 1 | 25.0 | 0 | 0.0 | 1 | 50.0 | 0 | 0.0 | 0 | 0.0 | 0 | 0.0 | 5 |
| Likely Benign | 3 | 8.6 | 4 | 36.4 | 2 | 50.0 | 0 | 0.0 | 0 | 0.0 | 0 | 0.0 | 0 | 0.0 | 0 | 0.0 | 9 |
| Uncertain Significance | 5 | 14.3 | 0 | 0.0 | 0 | 0.0 | 0 | 0.0 | 0 | 0.0 | 1 | 100.0 | 0 | 0.0 | 0 | 0.0 | 6 |
| Pathogenic | 8 | 22.8 | 4 | 36.4 | 0 | 0.0 | 2 | 100.0 | 1 | 50.0 | 0 | 0.0 | 0 | 0.0 | 1 | 100.0 | 16 |
| Likely Pathogenic | 4 | 11.4 | 1 | 9.1 | 1 | 25.0 | 0 | 0.0 | 0 | 0.0 | 0 | 0.0 | 1 | 100.0 | 0 | 0.0 | 7 |
| Conflicting Interpretation of Pathogenicity | 10 | 28.6 | 2 | 18.1 | 0 | 0.0 | 0 | 0.0 | 0 | 0.0 | 0 | 0.0 | 0 | 0.0 | 0 | 0.0 | 12 |
| Drug Response | 2 | 5.7 | 0 | 0.0 | 0 | 0.0 | 0 | 0.0 | 0 | 0.0 | 0 | 0.0 | 0 | 0.0 | 0 | 0.0 | 2 |

**Table (S6): Distribution of genetic variants according to** **clinical significance in Trastuzumab resistant patients**

| **Clinical significance** | **TP53**  **(n = 29)** | | **ATM**  **(n = 8)** | | **IDH1**  **(n = 3)** | | **RB1**  **(n = 3)** | | **GNAS**  **(n = 2)** | | **CDH1**  **(n = 1)** | | **MLH1**  **(n = 1)** | | **SMARCB1**  **(n = 1)** | | **SMO**  **(n = 1)** | | **VHL**  **(n = 1)** | | **Total**  **(n = 50)** |
| --- | --- | --- | --- | --- | --- | --- | --- | --- | --- | --- | --- | --- | --- | --- | --- | --- | --- | --- | --- | --- | --- |
|  | **No.** | **%** | **No.** | **%** | **No.** | **%** | **No.** | **%** | **No.** | **%** | **No.** | **%** | **No.** | **%** | **No.** | **%** | **No.** | **%** | **No.** | **%** |  |
| Benign | 0 | 0.0 | 0 | 0.0 | 0 | 0.0 | 0 | 0.0 | 0 | 0.0 | 0 | 0.0 | 1 | 100.0 | 0 | 0.0 | 0 | 0.0 | 0 | 0.0 | 1 |
| Likely Benign | 4 | 13.8 | 0 | 0.0 | 0 | 0.0 | 0 | 0.0 | 0 | 0.0 | 0 | 0.0 | 0 | 0.0 | 0 | 0.0 | 0 | 0.0 | 0 | 0.0 | 4 |
| Uncertain Significance | 2 | 6.9 | 4 | 50.0 | 0 | 0.0 | 0 | 0.0 | 0 | 0.0 | 1 | 100.0 | 0 | 0.0 | 0 | 0.0 | 0 | 0.0 | 0 | 0.0 | 7 |
| Pathogenic | 6 | 20.7 | 2 | 25.0 | 2 | 66.7 | 3 | 100.0 | 1 | 50.0 | 0 | 0.0 | 0 | 0.0 | 1 | 100.0 | 1 | 100.0 | 1 | 100.0 | 17 |
| Likely Pathogenic | 8 | 27.6 | 0 | 0.0 | 1 | 33.3 | 0 | 0.0 | 1 | 50.0 | 0 | 0.0 | 0 | 0.0 | 0 | 0.0 | 0 | 0.0 | 0 | 0.0 | 10 |
| Conflicting Interpretation of Pathogenicity | 7 | 24.2 | 2 | 25.0 | 0 | 0.0 | 0 | 0.0 | 0 | 0.0 | 0 | 0.0 | 0 | 0.0 | 0 | 0.0 | 0 | 0.0 | 0 | 0.0 | 9 |
| Drug Response | 1 | 3.4 | 0 | 0.0 | 0 | 0.0 | 0 | 0.0 | 0 | 0.0 | 0 | 0.0 | 0 | 0.0 | 0 | 0.0 | 0 | 0.0 |  | 0.0 | 1 |
| Unclassified | 1 | 3.4 | 0 | 0.0 | 0 | 0.0 | 0 | 0.0 | 0 | 0.0 | 0 | 0.0 | 0 | 0.0 | 0 | 0.0 | 0 | 0.0 | 0 | 0.0 | 1 |
